# SCOPe: Structural Classification of Proteins—extended, integrating SCOP and ASTRAL data and classification of new structures

**DOI:** 10.1093/nar/gkt1240

**Published:** 2013-12-03

**Authors:** Naomi K. Fox, Steven E. Brenner, John-Marc Chandonia

**Affiliations:** ^1^Physical Biosciences Division, Lawrence Berkeley National Laboratory, Berkeley, CA 94720, USA and ^2^Department of Plant and Microbial Biology, University of California, Berkeley, CA 94720, USA

## Abstract

Structural Classification of Proteins—extended (SCOPe, http://scop.berkeley.edu) is a database of protein structural relationships that extends the SCOP database. SCOP is a manually curated ordering of domains from the majority of proteins of known structure in a hierarchy according to structural and evolutionary relationships. Development of the SCOP 1.x series concluded with SCOP 1.75. The ASTRAL compendium provides several databases and tools to aid in the analysis of the protein structures classified in SCOP, particularly through the use of their sequences. SCOPe extends version 1.75 of the SCOP database, using automated curation methods to classify many structures released since SCOP 1.75. We have rigorously benchmarked our automated methods to ensure that they are as accurate as manual curation, though there are many proteins to which our methods cannot be applied. SCOPe is also partially manually curated to correct some errors in SCOP. SCOPe aims to be backward compatible with SCOP, providing the same parseable files and a history of changes between all stable SCOP and SCOPe releases. SCOPe also incorporates and updates the ASTRAL database. The latest release of SCOPe, 2.03, contains 59 514 Protein Data Bank (PDB) entries, increasing the number of structures classified in SCOP by 55% and including more than 65% of the protein structures in the PDB.

## BACKGROUND

Nearly all proteins have structural similarities with other proteins and, in many of these cases, share a common evolutionary origin. The Structural Classification of Proteins (SCOP) database (1–4), which marks its 20th anniversary this year, aimed to provide a detailed and comprehensive description of the structural and evolutionary relationships between all proteins of known structure. As such, it provides a broad survey of known protein folds, detailed information about the close relatives of any particular protein and a framework for future research and classification.

By analogy with taxonomy, SCOP was created as a hierarchy of several levels where the fundamental unit of classification is a ‘domain’ in the experimentally determined protein structure. The hierarchy of SCOP domains comprises the following levels: ‘Species’ representing a distinct protein sequence and its naturally occurring or artificially created variants; ‘Protein’ grouping together similar sequences of essentially the same functions that either originate from different biological species or represent different isoforms within the same species; ‘Family’ containing proteins with similar sequences but typically distinct functions and ‘Superfamily’ bridging together protein families with common functional and structural features inferred to be from a common evolutionary ancestor. Near the root, the basis of classification is purely structural: structurally similar superfamilies are grouped into ‘Folds’, which are further arranged into ‘Classes’ based mainly on their secondary structure content and organization.

The ASTRAL compendium (5–7) is a collection of software and databases, partially derived from SCOP, that aid research into protein structure and evolution. ASTRAL provides sequences and coordinate files for all SCOP domains, as well as sequences for all Protein Data Bank (PDB, 8) chains that are classified in SCOP. Chemically modified amino acids are translated back to the original sequence, and sequences are curated to eliminate errors resulting from the automated parsing of PDB files. Because the majority of sequences in the PDB are very similar to others, ASTRAL provides representative subsets of proteins that span the set of classified protein structures or domains while alleviating bias toward well-studied proteins. The highest quality representative in each subset is chosen using AEROSPACI scores (7), which provide a numeric estimate of the quality and precision of crystallographically determined structures.

The SCOP database was first released in December 1994 and contained all 3091 entries in the PDB at that time (1). Table 1 provides a history of all stable SCOP releases, including the number of months required to curate each release. The last comprehensive release was SCOP 1.71, released in October 2006. The increase in time required to fully classify each comprehensive SCOP release, shown in Table 1, highlights the need to abet manual classification with automated methods. Although SCOP was fully manually curated prior to version 1.73, an automated classification method, discussed later in this manuscript, was introduced in SCOP 1.73. SCOP levels above ‘Species’ were hand-curated in all versions of SCOP; these include the ‘Superfamily’ level, in which human expertise was used to annotate many remote homologs that cannot be reliably identified as homologous by current computational methods. Development of the SCOP 1.x series concluded with SCOP 1.75, released in June 2009. It classified 38 221 PDB entries, more than 70% of the PDB structures available at that time. However, due to increasing growth of the PDB, SCOP 1.75 now covers fewer than half of the protein structures that are currently available.

**Table 1.** SCOP and SCOPe growth and benchmarking

Release	Freeze date	Release date	Months to release	Total PDB entries	Total PDB entries classified	New PDB entries used in benchmark	PDB deposition rate per month	Percent of new entries classifiable by current automated method
SCOP 1.55	2001–03	2001–07	4	13 300	13 228	n/a	258	n/a
SCOP 1.57	2001–10	2002–01	3	14 825	14 736	1508	275	49
SCOP 1.59	2002–03	2002–05	2	16 057	15 985	1249	270	47
SCOP 1.61	2002–09	2002–11	2	17 498	17 411	1426	304	51
SCOP 1.63	2003–03	2003–06	3	19 036	18 951	1540	351	50
SCOP 1.65	2003–08	2003–12	4	20 699	20 619	1668	374	51
SCOP 1.67	2004–05	2005–02	9	24 131	24 036	3417	436	52
SCOP 1.69	2004–10	2005–07	9	26 101	25 972	1936	454	46
SCOP 1.71	2005–01	2006–10	21	27 821	27 599	1627	474	45
SCOP 1.73	2007–09	2007–11	2	44 169	34 494	6895	593	58
SCOP 1.75	2009–02	2009–06	4	53 830	38 221	3727	632	48
SCOPe 2.01 (formerly 1.75A)	2012–02	2012–03	1	76 528	49 219	n/a	775	n/a
SCOPe 2.02 (formerly 1.75B)	2012–11	2013–01	2	83 643	49 674	n/a	816	n/a
SCOPe 2.03 (formerly 1.75C)	2013–08	2013–10	2	90 812	59 514	n/a	n/a	n/a

The number of new entries added in each release of SCOP that used stable identifiers. For each release, the ‘freeze date’, or date at which no new PDB entries were to be classified in the release, is given. In practice, some entries released just after the freeze date were sometimes included. The total number of PDB entries that contained protein structures, were not obsolete as of the freeze date, or which were included in each release, is given, as well as the number of PDB entries that were included in each release. Release 1.71 was the most recent comprehensive SCOP release (i.e. one in which nearly all PDB entries available prior to the freeze date were classified). The average rate at which PDB entries were deposited each month is also given, measured over the 6 months before and after (if applicable) the freeze date.

In this article, we describe SCOP—extended (SCOPe), a database that extends SCOP 1.75, with the aim of providing ongoing classification of new PDB structures in the context of the SCOP hierarchy, backward compatibility with SCOP, manual correction of errors and stable releases suitable for benchmarking. More than 800 new structures are now being deposited to the PDB in an average month (8), too many for a workflow based entirely on manual curation to keep pace. We have therefore developed new methods for automatically classifying structures similar to those already in SCOP, and we have benchmarked it rigorously with the goal of achieving accuracy comparable to fully hand-curated SCOP releases (i.e. through SCOP 1.71). However, these methods do not classify structures dissimilar to those already classified manually. New releases of ASTRAL are now derived from the SCOPe classification, and ASTRAL data are integrated into the SCOPe website. SCOPe also includes data from all previous releases of SCOP and ASTRAL that feature stable identifiers (i.e. since SCOP 1.55) to facilitate web-based comparison of releases of SCOP and SCOPe. SCOPe releases 2.01, 2.02 and 2.03 do not add additional manually curated entries at the ‘Family’ level or above. Rather, domains from new protein structures are classified into the manually curated hierarchy of SCOP 1.75, and many existing SCOP 1.75 domains were corrected or moved. Statistics on all SCOP and SCOPe releases, summaries and a full history of changes and other information are available from the SCOPe website (http://scop.berkeley.edu/) together with copies of our relational (MySQL) database and parseable files containing all SCOPe, SCOP and ASTRAL data.

## BACKWARD COMPATIBILITY

To facilitate use of SCOPe data by SCOP and ASTRAL users, we provide SCOPe and ASTRAL data in parseable files in the same formats as the previous SCOP and ASTRAL releases. SCOPe uses the same stable identifiers (e.g. sunid, sid, sccs) as were used for prior releases of SCOP and ASTRAL (3,5,6), and the same protocols previously used to assign new SCOP and ASTRAL identifiers are currently being used in SCOPe. A history of all changes between consecutive releases of SCOP and SCOPe is available on the SCOPe website.

## ADDING NEW ENTRIES AND CORRECTING ERRORS IN SCOP

**Figure 1.**
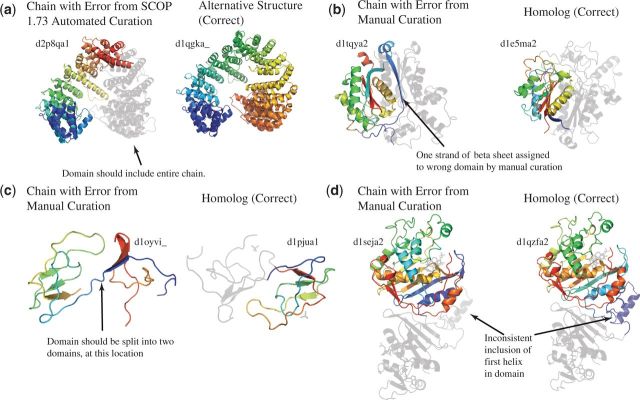
Errors identified during benchmarking. We detected errors in 70 manually curated domains by running benchmarking and manually inspecting predicted domains that did not sufficiently match the manually annotated domains. These errors in domain boundaries in multi-domain chains were manually fixed in SCOPe 2.03. We also detected and fixed inconsistencies in 5054 domains that had been predicted and classified with the SCOP 1.73 automated method. We review some of the types of errors detected. (**a**) The SCOP 1.73 automated method used to predict domain d2p8qa1 had included approximately half the residues in the chain. This was inconsistent with all other manually curated entries in its species-level clade that included the entire chain. (**b**) A strand of beta sheet was included in the d1tqya2 domain by manual curation. (**c**) All of chain I from 1oyv had been placed into a single domain. (**d**) The manually curated domain d1seja2 excluded the first helix in the chain.

Using our new automation procedure (described in the next section), we have added 21 293 PDB files to SCOPe that were not classified in SCOP 1.75 (∼23% of the PDB). We have also modified some previous entries that were manually curated or classified via a previous automated method introduced in SCOP 1.73 (4). Based on careful examination of differences between automated classification and manually curated domains, we detected and fixed errors in 70 manually curated domains in SCOP (out of 105 detected with differences), of which 62 were changed by more than 10 residues. The fraction of manually classified domains in SCOP 1.75 with substantial errors we could detect was 0.08% (62 of 80 140), reflecting the extremely high accuracy of manual classification in the SCOP database. Typical examples of common errors are shown in Figure 1. Often these errors were the result of a typo during manual curation and would result in the improper inclusion or exclusion of a region of sequence from a domain. We reassigned domain boundaries to 5054 domains that had been classified with the SCOP 1.73 automated method. The vast majority of these were small changes, but 231 differed substantially, requiring a domain to be split into two or merged with another, or its boundary to be changed by more than 10 residues. This also represents a low error rate: 0.8% (231 of 30 660), although it is roughly 10 times higher than the error rate for manually curated domains. We also noticed a number of both manually and automatically curated domains that had been classified in the wrong ‘Protein’ or ‘Species’ entry. We have corrected 252 manually curated domains and 6285 previous automatically classified domains.

## AUTOMATED CLASSIFICATION METHODS

We have implemented a new automated classification algorithm, based on sequence similarity to previously classified entries, and benchmarked this method against all stable releases of SCOP from 1.57 to 1.75.

An automated method for classifying new PDB entries based on previously classified entries was introduced in SCOP 1.73 (4); however, rigorous benchmarking was not used to validate this method. We have found inconsistencies between manually curated domains and those determined with this method. Figure 1 includes an example of such an inconsistency. Other methods such as SUPERFAMILY (9,10) and SCOPmap (11) have been developed to automatically classify new PDB structures in the context of SCOP and have the advantage of operating on proteins somewhat dissimilar from those already classified. However, when validated against manually curated domains, these methods have error rates that are too high to be suitable for incorporating their results directly into SCOP. For example, SCOPmap reports a 5% error rate in benchmarking experiments. Our aim in developing a new automated classification algorithm was to maximize the number of domains that could be classified with an error rate indistinguishable from manual curation (see discussion of manual curation error rate above).

The SCOP automated curation pipeline has been completely rewritten in SCOPe. We are replacing all domains that were predicted with the less accurate SCOP 1.73 automated method with newer predictions. Over the course of benchmarking our new method, we found the method would have been able to automatically assign domains for approximately half of the PDB entries that were manually curated in prior versions of SCOP, with no substantial differences (i.e. >10 residue difference in any domain boundary) between the automatically assigned and manually curated domain boundaries. Over the course of benchmarking, we found some differences that, upon expert examination, were determined to be the result of manual curation errors in prior versions of SCOP. These are discussed later in the manuscript and were corrected in SCOPe 2.03.

One of the main challenges in automatically assigning domains based on previously classified homologs is that different homologs may have different observed residues (i.e. those present in the ATOM records of PDB data). SCOP domains in a structure are defined relative to the observed residues. It is sometimes challenging to correctly assign observed residues in a new structure that were not observed in a previously classified structure. Another challenge is in variance in manually curated boundaries. We found that in multi-domain chains with long linker regions, the exact boundaries between domains within a family can differ substantially. This is because fully manually curated releases of SCOP (those before 1.73) aimed to classify every residue in each PDB structure, even though it is sometimes unclear to which, if either, of the adjacent domains residues in a linker region should be assigned.

We now describe our algorithm for predicting domains and classifying them in the SCOP hierarchy. We first create a BLAST database containing the SEQRES-based sequences for each domain in SCOP and SCOPe. Then, for each newly released PDB chain, we BLAST its SEQRES sequence against the domain sequence database. BLAST performs local alignment, returning the start and end of the alignment (the ‘hit’) for the query sequence and the target sequence, as well as the E-value. We collect only the BLAST alignments where the E-value is at least as significant at 10^−^^4^ and the alignment covers most of the target domain (defined as missing at most 10 residues from each end). We group the alignments by the PDB chain that the targets belong to and rank the groups by the total number of residues covered by the hits on the query chain. We then use the top-ranking group of BLAST-based alignments to annotate domains in the query sequence. If the nearest PDB chain end or gap in ATOM records is within 10 residues of an alignment end, we extend the domain to include these residues. The purpose of extending the BLAST hit is to classify every observed residue in the chain; the 10-residue limitation makes it very unlikely that the extension will include a new domain. If the BLAST boundaries are outside the observed residues in the query chain, the assigned domain is shortened to include only observed residues.

After making predictions for each chain, we applied a set of criteria to determine whether each domain prediction was high confidence (i.e. sufficiently accurate to be included in SCOPe without further manual inspection). We first exclude from high confidence those chains that are low-resolution (3 Å resolution or above), ribosomal or synthetic (due to those being classified outside the first seven classes of SCOP), or those that are homologous to genetic domains classified in SCOP (due to the difficulty of correctly automating predictions that include multiple PDB chains).

In cases where an entire PDB chain was predicted to be a single SCOPe domain, the prediction was deemed high confidence if the target domain also comprised its entire PDB chain. For PDB chains that were divided into multiple SCOPe domains, additional criteria were used to determine whether the predicted domains were high confidence. First, we restricted the chains whose predictions we placed in the high confidence set to those which either (i) had 100% sequence identity with the target chain used to make the predictions or (ii) had exactly two domains, each composed of only one contiguous region of residues. We plan to extend the method to three or more domain chains and multi-region domains in the future. Second, if any region found in the ATOM records of the chain was longer than 10 residues and not assigned to any domain, we removed the chain from the high-confidence set. Third, we required the BLAST hits used for the two domains in the chain not be to the same target SCOP domain (on the theory that domain duplications are more likely to require a specialized algorithm or manual inspection to ensure no structural changes such as domain swapping).

To fully classify these predictions in the SCOP hierarchy, we also had to assign levels below ‘Superfamily’. Based on our benchmarking, we developed heuristic rules to classify domains at the ‘Family’, ‘Protein’ and ‘Species’ levels. In cases where the protein or family could not be reliably matched to an existing SCOP entity, we created a new protein or family called ‘automated matches’ rather than risking inaccurate classification.

Thus far our focus has been on classifying chains that have high sequence similarity with previously classified chains in SCOP and therefore have relied solely on sequence data. The addition of structural information should expand the set of domains that can be classified with high confidence in the future.

## BENCHMARKING RESULTS

**Figure 2.**
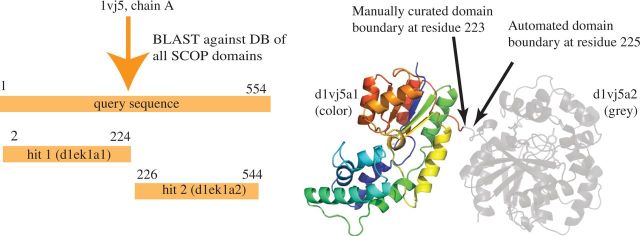
Automated curation example. This figure depicts an example of applying the automated method for domain prediction and classification to 1vj5, chain A, released on 2004-04-27. We attempted to automatically classify it into SCOP 1.67, based only on domains defined in SCOP 1.65. 1vj5A has 554 residues, of which residues 2-547 are observed (found in the ATOM records in PDB data). Two significant BLAST hits were found to the classified chain 1ek1A, which has a distinct sequence from 1vj5A but also has 554 residues, of which residues 4-19, 48-66 and 90-544 are observed. The two BLAST hits include residues 2-224 and 226-544 in 1vj5A. The final predicted domains in 1vj5A are 2-225 and 226-547. The manually annotated domains for 1vj5A are 2-223 and 224-547. Since the end of each predicted domain differs from the manually annotated domain by at most 10 residues, this domain prediction is deemed to fall within the error tolerance for validation.

To validate the new automated method, we performed benchmarking against all SCOP releases with stable identifiers (i.e. releases 1.55–1.75). All PDB entries that were added between each pair of consecutive releases were automatically classified based on the earlier release and compared with the manually curated domains in the subsequent release. A predicted domain was considered identified and classified correctly if it was placed in the correct superfamily and its boundaries differed from the manually curated boundaries by no more than 10 residues. Table 1 lists the fraction of PDB entries that could be classified using this method for each SCOP release. A non-trivial example of automated classification is shown in Figure 2. Our method predicted 20 048 domains that matched manually curated domains within the 10-residue error tolerance and predicted 105 domains that differed by more than 10 residues. We reviewed all differences of more than 10 residues, and found they were either the result of errors in SCOP 1.75 (discussed above) or in linker regions in which neither the manually curated domain nor the prediction could be determined to be more accurate. We also compared domains that had been added by the previous automated method (4), which had added 30 852 new domains in total to versions 1.73 and 1.75. We compared domains predicted by our new method to those added by the old method. Of the 15 660 domain pairs compared (the new automated method is more conservative than the old one, and therefore fewer domains are classified), 125 were found to have domain boundaries that differed by more than 10 residues (this is lower than the 231 such domains we corrected in SCOPe, discussed above, because the benchmark did not include additional SCOPe domains based on manually curated domains from SCOP 1.75). These differences were also the result of errors in the previous automated method or ambiguous linker regions.

## NEW WEBSITE

The SCOPe website offers a modest redesign of the SCOP website, presenting all SCOPe, SCOP and ASTRAL data through a single, unified web interface. Many objects that were difficult to find in the original website, such as the change history, are now available under tabs. Thumbnail images were automatically generated to show each domain on its own and in several structural contexts, and these are displayed as part of the browser. A fully JavaScript JSmol-based viewer was added to enable visitors to view domains in three-dimensions in isolation, in context of the chain or in context of the entire PDB structure. The SCOPe website can display data from all versions of SCOPe, SCOP and ASTRAL since release 1.55. All data are stored in a relational (MySQL) database, which is also available for download.

## STABLE AND PERIODIC UPDATES

Stable releases will potentially include manual curation of the SCOPe hierarchy, correction of errors in previously classified domains or changes to our classification workflow (e.g. validation of our automated sequence-based classification protocol with structure comparison). However, in an effort to stay more closely synchronized with the PDB, we also plan to supplement these stable releases with periodic updates (approximately monthly). Our infrastructure automatically imports and classifies new PDB files on a weekly basis. Starting with SCOPe 2.02, we have begun to release periodic updates that add newly released PDB entries to the SCOPe classification. These periodic updates add new PDB entries to the current release, without affecting any previously classified domains. The newly classified entries are visible in the web interface and in downloadable files such as the SCOP-compatible parseable files and MySQL database. Sequences for the newly added chains and domains will not be added to the ASTRAL representative subsets until the subsequent stable release of ASTRAL.

The periodic updates are not intended to replace stable releases; because the latter are commonly used for benchmarking, both will be available for download through the SCOPe website. Stable releases will be assigned version numbers in the current format (explicitly labeled stable on the website and downloadable files, e.g. 2.03-stable), while updates to stable releases will be named according to the most recent stable version appended with the release date (e.g. 2.03-2013-12-01).

## FUNDING

This work is supported by the National Institutes of Health (NIH) [R01-GM073109] through the US Department of Energy under Contract No. DE-AC02-05CH11231. Funding for open access charge: NIH [R01-GM073109].

*Conflict of interest statement.* None declared.
